# Establishment and effectiveness evaluation of pre-test probability model of coronary heart disease combined with cardiopulmonary exercise test indexes

**DOI:** 10.1038/s41598-023-41884-x

**Published:** 2023-09-29

**Authors:** Si Xu Liu, Sheng Qin Yu, Kai Jing Yang, Ji Yi Liu, Fan Yang, Ye Li, Chang Li Yao, Guang Sheng Zhao, Feng Zhi Sun

**Affiliations:** 1 Heart Center, Affiliated Zhongshan Hospital of Dalian University, No.6 Jie Fang Street, Zhongshan District, Dalian, 116001 Liaoning Province China; 2 Minimally invasive interventional diagnosis and treatment center, Affiliated Zhongshan Hospital of Dalian University, No.6 Jie Fang Street, Zhongshan District, Dalian, 116001 Liaoning Province China

**Keywords:** Cardiology, Diseases, Medical research, Risk factors, Signs and symptoms

## Abstract

To establish a pre-test probability model of coronary heart disease (CHD) combined with cardiopulmonary exercise test (CPET) indexes and to compare the clinical effectiveness with Duke clinical score (DCS) and updated Diamond-Forrester model (UDFM), thus further explore the predictive value. 342 cases were used to establish the prediction model equation and another 80 cases were used to verify the effectiveness. The patients were divided into CHD group (n = 157) and non-CHD group (n = 185) according to coronary artery stenosis degree >50% or not. Combining DCS and UDFM as reference models with CPET indexes, a multivariate logistic regression model was established. The area under the ROC curve of the three models were calculated to compare the predictive effectiveness. There were significant differences in gender, chest pain type, myocardial infarction history, hypertension history, smoking, pathological Q wave and ST-T change between two groups (P < 0.01), as well as age, LVEF, heart rate at anaerobic domain, peak oxygen uptake in kilograms of body weight, percentage of peak oxygen uptake to the predicted value, the oxygen uptake efficiency slope and carbon dioxide ventilation equivalent slope (P < 0.05). Multivariate analysis showed gender, age, chest pain type, myocardial infarction history, hypertension history, smoking, pathological Q wave, ST-T change, and peak oxygen pulse were independent risk factors of CHD. The pre-test probability model of CHD combined with CPET indexes has good distinguish and calibrate ability, its prediction accuracy is slightly better than DCS and UDFM, which still needs to be verified externally in more samples.

## Introduction

Currently, cardiovascular disease has the highest morbidity and mortality rates among non-communicable diseases worldwide, and it is increasing year by year. The early diagnosis and treatment of coronary heart disease (CHD) are particularly important^1^. Coronary angiography (CAG), intravascular ultrasound (IVUS) and optical coherence tomography (OCT) are widely used in clinical practice as the "gold standard" for the diagnosis of CHD. But these tests are still essentially invasive coronary angiography (ICA). A large study of more than 30,000 patients with suspected stable angina found that 51% of women and 33% of men did not have significant coronary stenosis^2^.

The advantages of noninvasive testing over ICA are clear. Pre-test probability (PTP) is recommended in both European and United States CHD guidelines as an indicator to guide clinical decision-making in patients with suspected CHD^3–6^. The probability for predicting obstructive CHD is obtained through history taking, physical examination, and electrocardiography in suspected patients, and non-invasive evaluation is performed on suspected patients before ICA validation, thereby increasing the positive rate of ICA diagnosis and reducing the mistake diagnostic rate of CHD. Of which, the American Heart Association/American College of Cardiology (AHA/ACC) recommends the use of the Duke clinical score (DCS) model to estimate the pre-test probability of suspected CHD^5,6^, with the DCS markers being gender, age, type of chest pain, history of infarction, history of diabetes, hyperlipidemia, history of smoking, pathological Q waves, and ST-T changes. The European Society of Cardiology (ESC) recommends the calculation of a more convenient updated Diamond-Forrester model (UDFM) to guide clinical decision-making^5,6^. Some studies have independently verified the DCS model in Japanese population^7^, and it was found that the DCS model overestimated the prevalence of the local population by about 2.4 times. This phenomenon may be due to the fact that the model based on the high prevalence population will overestimate the true prevalence of the low prevalence population, and the CHD risk factors of the European and American populations are different from those of the Asian population.

Non-invasive test items for CHD that are not currently included in the DCS and UDFM models include blood biochemistry, echocardiography, cardiopulmonary exercise testing (CPET), computed tomographic coronary angiography (CTCA), and nuclear myocardial perfusion imaging. A small number of domestic and international studies have explored the improvement and upgrading of the guideline-recommended model^8,9^. Edlinger et al.^8^ conducted Diamond-Forrester model validation on 4888 patients who underwent ICA. After combining blood biochemical indicators with the initial model, the area under the ROC curve (AUC) of the model could be improved from 0.69 to 0.72 (P < 0.05). Combining estrogen level, gestational diabetes mellitus and UDFM model^9^, the AUC of the model can be increased from 0.61 to 0.71 (P < 0.05). These above studies suggest that models can be constructed to improve predictive accuracy by incorporating more specific risk factors.

Although CPET does not provide hemodynamic evidence, it is more cost-effective compared to ICA. And it can reflect coronary spasm and microangiopathy. Ekelund et al.^10^ found a 2–fivefold increase in the incidence of CHD or mortality for every 2-unit decrease in peak oxygen uptake (peak VO_2_) from the follow-up in 4276 men. Alberto et al.^11^ found that carbon dioxide ventilation equivalent slope (VE/VCO_2_ slope) could be used to predict coronary perfusion defects in patients with suspected CHD. Ganesananthan et al.^12^ found that the oxygen pulse curve platform can detect the severity of myocardial ischemia and predict the prognostic efficacy of interventional therapy in patients with monovascular lesions.

In conclusion, the current general DCS and UDFM models and the upgraded models based on the two models do not include CPET indexes for multifactorial analysis, and relevant studies at home and abroad have proved the diagnostic value of CPET indexes for CHD. In this study, we propose to establish a pre-test probability model of CHD combined with CPET indexes, in order to compare the clinical effectiveness of the established model with that of the DCS and the UDFM models, and to explore the predictive value of the established model for CHD.

## Subjects and methods

### Research subjects

A total of 422 inpatients with suspected CHD who underwent ICA and CPET from January 2017 to January 2021 in Affiliated Zhongshan Hospital of Dalian University were enrolled, of which 342 cases were used to establish the prediction model in random order and another 80 cases were used to verify the effectiveness of the prediction model. The patients were divided into CHD group (n = 157) and non-CHD group (n = 185) according to whether the degree of coronary artery stenosis was greater than 50% (at least one main coronary artery or its main branch vessels). The study complied with medical ethics guidelines and all enrolled patients signed the informed consent form.

#### Inclusion criteria

1. Patients older than 18 years but younger than 80 years;
2. Patients who admitted for chest pain or suspected CHD;
3. Patients who are clinically stable and can meet the requirements of the CPET.

#### Exclusion criteria

1. Patients with non-anginal chest pain or chest pain of indistinguishable type;
2. Patients with unstable clinical condition or in acute exacerbation;
3. Patients with acute coronary syndrome, acute heart failure, malignant arrhythmia, chronic obstructive pulmonary disease, cardiomyopathy, abnormal neuromuscular strength, peripheral vascular system disease, and other diseases that affect the test results.

### Research methods

#### General information

Gender, age, type of chest pain, history of myocardial infarction, history of hypertension, hyperlipidemia, history of diabetes, history of smoking, history of alcoholism, family history, and body mass index of patients were included as observation indicators, while pathological Q-wave and ST-T changes on ECG, left ventricular ejection fraction on cardiac ultrasound, and CPET indexes were recorded.

Definition of chest pain: 1. Chest pain induced by exertion, physical exercise or emotional excitement; 2. The pain is located in the retrosternal or precordial region; 3. The pain can be relieved within a few minutes after rest or with nitrate drugs. If all three of the above items are met at the same time, the patient is considered to have typical angina pectoris. If one or two of the above items are met, the patient is considered to have atypical angina pectoris. If none of the above item is met, the patient is excluded.

ECG diagnosis: pathological Q-wave time frame ≥ 40 ms; Q-wave amplitude > 1/4 simultaneous lead R-wave; Q-wave appears in the lead where it should not be. ST-T changes: T-wave inversion or ST-segment downshift ≥ 0.1 mV on 12-lead ECG.

Based on these definitions, two cardiologists performed independent manual confirmation of the chest pain type of patients and the ECG features using the electronic medical record system. When the judgment is inconsistent, discuss and decide together. The definitions of other data were discriminated with reference to international uniform standards.

#### DCS and UDFM models

The DCS markers being gender, age, type of chest pain, history of infarction, history of diabetes, history of hyperlipidemia, history of smoking, pathological Q-wave, and ST-T changes, and the model formula is

1 $${\text{PTPDCS}} = {1}/\left( {{1} + {\text{ea}}} \right),$$

where a = −(−7.376 + 0.1126 × age-0.328 × gender-0.0301 × age × gender + 2.581 × typical angina pectoris + 0.976 × atypical angina pectoris + 1.093 × history of infarction + 1.213 × pathological Q-wave + 0.741 × history of infarction × pathological Q-wave + 2.596 × smoking + 0.694 × diabetes + 1.845 × hyperlipidemia + 0.637 × ST-T changes—0.0404 × age × smoking—0.0251 × age × hyperlipidemia + 0.550 × gender × smoking). Categorical variable assignment (0 for none, 1 for yes, 0 for male, 1 for female).

UDFM markers were gender, age, and type of chest pain. The model formula is

2 $${\text{PTPUDFM}} = {1}/\left( {{1} + {\text{eb}}} \right),$$

where b = −(−4.37 + 0.04 × age + c + d), c = 1.34 (male) or 0 (female), d = 1.91 (typical angina pectoris) or 0.64 (atypical angina pectoris) or 0 (non-anginal chest pain), for patients aged 71–80 years, statistical calculations were performed with reference to 70 years (the upper limit age of DCS was 70 years, and there were 8 cases in the sample), and the relevant clinical data of the patients were collected through the electronic medical record system.

#### CPET

The SCHILLER CS-200 cardiopulmonary exercise test system was used. The cardiopulmonary indexes of the patients at rest and during exercise were measured. The exercise volume was set with reference to the patient's age, heart rate and maximum Borg respiratory index. Each cardiopulmonary exercise parameter was recorded after 3 min of rest, after 3 min of no-load warm-up, after the initial to symptom-limited extreme amount of exercise, and the cardiopulmonary exercise parameters within 5 min of the initial recovery. The exercise test was terminated when the patients showed symptoms such as angina, fatigue, dyspnea, ECG changes, arrhythmia, lower limb pain, dizziness, and unsteadiness in standing.

#### Echocardiography

The PHILIPS EPIQ 7C color Doppler ultrasound system was used to measure and calculate the left ventricular ejection fraction (LVEF) of the patients by the Simpson method.

#### Coronary angiography (CAG)

GE Innova IGS 520 digital subtraction angiography machine was used and standardized operated by a cardiologist, and Seldinger's method was used to puncture the radial artery. Sending angiography catheter and injecting contrast agent, and then left and right coronary angiograms were performed with standard multi-angle body projection. The imaging records were interpreted and reported independently by 2 specialized interventional cardiology physicians, and in case of inconsistent results, the decision was made by a third physician in collaboration. The maximum stenosis degree of the lesion in each body position was taken and reported.

#### Statistical analysis

**Figure 1:**
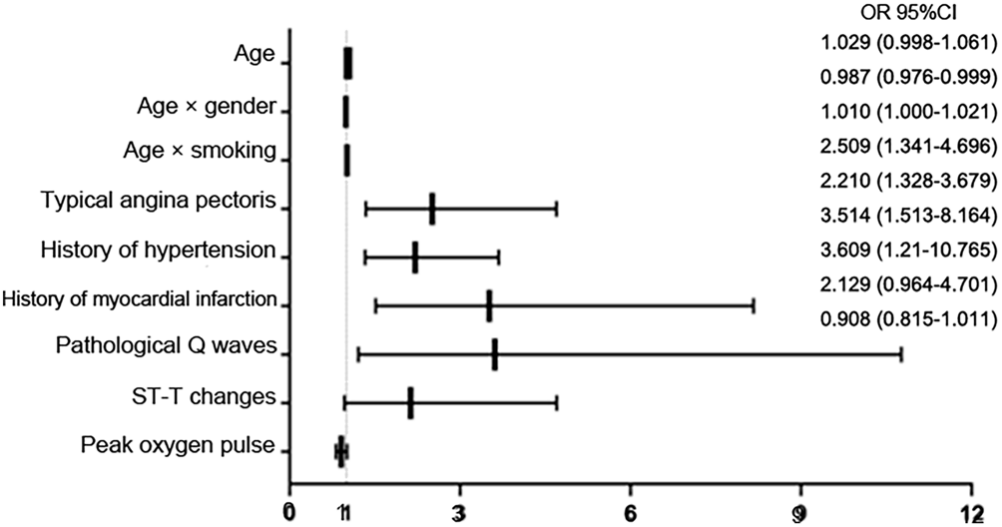
Forest plot and odds ratios (OR) value of CHD related factors.

**Figure 2:**
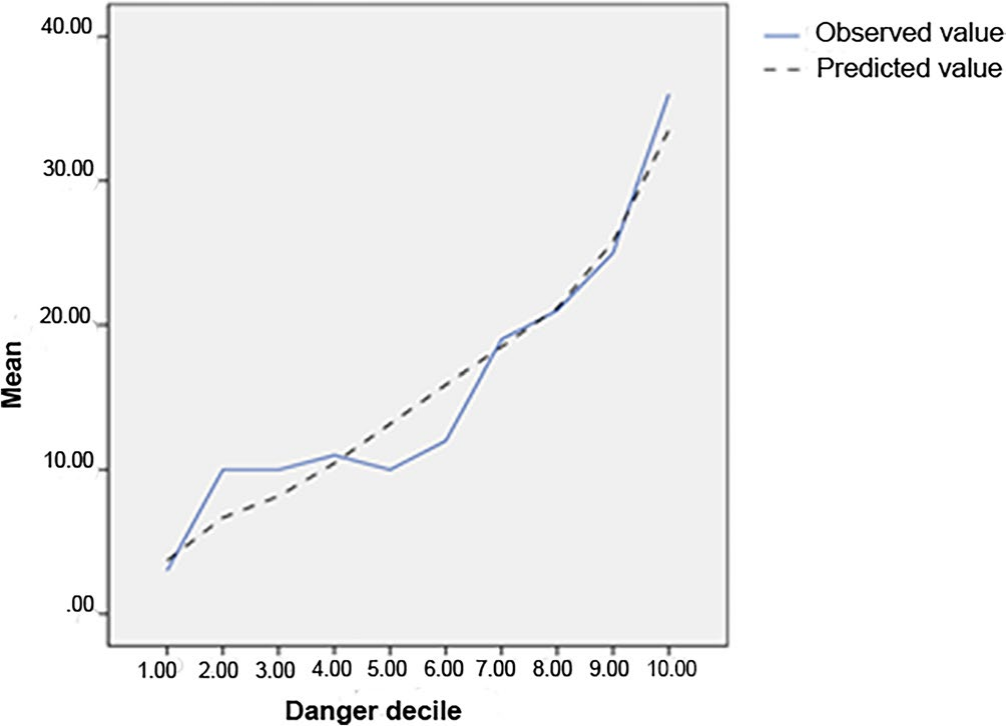
Line plot of the actual observed values versus the model predicted values.

**Figure 3:**
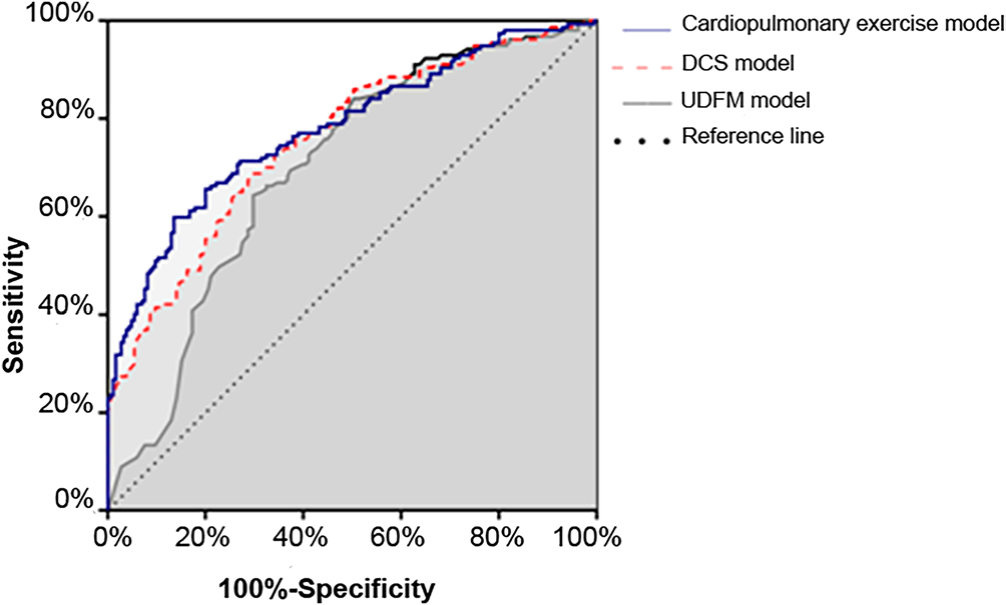
ROC curves of predictive models in modeling sample.

**Figure 4:**
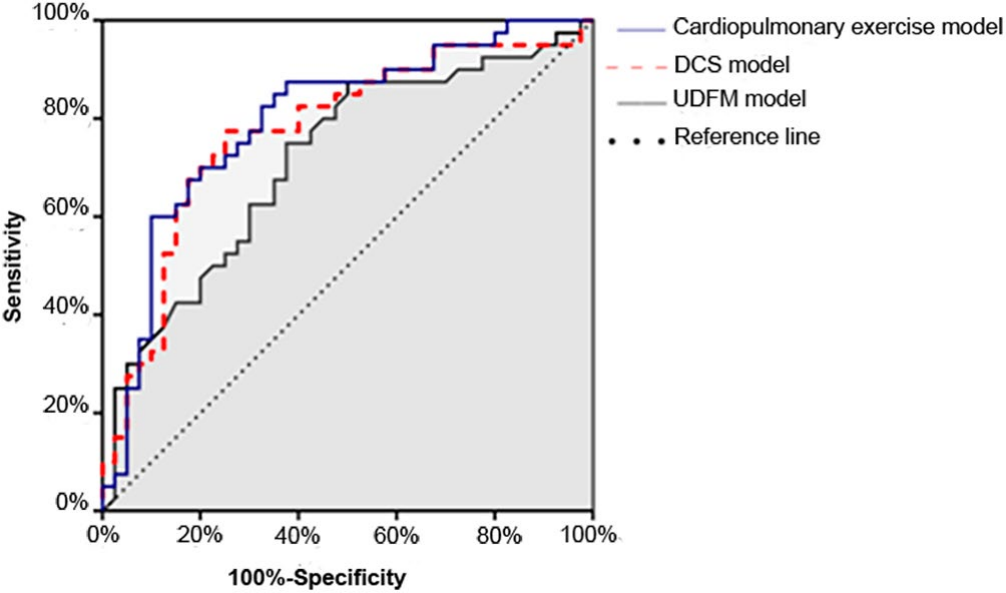
ROC curves of predictive models in validation sample.

SPSS 20.0 software was used for statistical analysis, and quantitative data were described as mean ± standard deviation or median (P25, P75), Two independent samples t-test or Wilcoxon rank sum test were used for comparison between groups. Qualitative data were described as frequencies (%), and the χ^2^-test or Fisher's exact probability method were used for comparison between groups. Univariate analysis was used for the initial screening of relevant risk factors, and ROC curves were drawn to determine the single factors critical value, sensitivity, specificity and Youden index. All variables with statistical differences in the single factor analysis were screened, and the most representative indicators were selected to reduce multicollinearity for indicators that are obviously related to each other (such as oxygen uptake, heart rate, and oxygen pulse). At the same time, variables (such as age × gender) that have been proved to have interaction in the DCS model were included, and the coronary angiography results were used as dependent variables for Logistic multiple regression analysis. Stepwise progressive regression maximum partial likelihood analysis was used to eliminate variables with low significance (P > 0.1), and the significant risk factors and the OR values for predicting CHD were determined (Table 1 and Fig. 1). Significant risk factors predicting coronary heart disease and the OR values were determined, the C-statistic and Hosmer–Lemeshow index were calculated to assess the consistency of the model in predicting coronary heart disease (Table 2 and Fig. 2). The cardiopulmonary model, DCS model and UDFM model were used to calculate the probability of CHD in each patient, and the ROC curves of the three models were drawn to predict coronary heart disease under the modeling sample and the validation sample with PTP as the test variable and the coronary angiography result as the state variable, and the model efficacy was evaluated by comparison (Figs. 3 and 4).

**Table 1:** Results of multivariate logistic regression analysis.

Factors	Regression coefficients (β)	Standard Error	Z value	P value	OR value (95% CI)	Tolerance	VIF
Age	0.029	0.016	3.322	0.068	1.029 (0.998–1.061)	0.888	1.127
Age × Gender	-0.013	0.006	4.641	0.031	0.987 (0.976–1.061)	0.526	1.901
Age × Smoking	0.010	0.005	3.610	0.057	1.010 (1.00–1.061)	0.748	1.338
Typical angina pectoris	0.920	0.320	8.274	0.004	2.509 (1.341–1.061)	0.928	1.077
History of myocardial infarction	1.257	0.430	8.538	0.003	3.514 (1.513–1.061)	0.671	1.490
History of hypertension	0.793	0.260	9.312	0.002	2.210 (1.328–1.061)	0.924	1.082
Pathological Q wave	1.283	0.558	5.297	0.021	3.609 (1.210–1.061)	0.691	1.447
ST-T changes	0.755	0.404	3.492	0.062	2.129 (0.964–1.061)	0.853	1.173
Peak oxygen pulse	-0.097	0.055	3.092	0.079	0.908 (0.815–1.061)	0.660	1.514
Constants	-2.337	1.211	3.724	0.054

**Table 2:** Model consistency.

Predictive models	Coronary angiography		Total
	CHD group	non-CHD group	
CHD	102	37	139
Non-CHD	55	148	203
Total	157	185	342 (0.731)

### Ethical approval

The study was approved by the Ethics Committee of Affiliated Zhongshan Hospital of Dalian University.

### Consent to participate

Informed consent was obtained from all individual participants included in the study.

## Results

### Comparison of qualitative data between CHD group and non-CHD group

There were statistically significant differences in gender, type of chest pain, history of myocardial infarction, history of hypertension, history of smoking, pathological Q wave, and ST-T changes between the two groups (P < 0.01), while the other indexes were not statistically significant (Table 3).

**Table 3:** General information of patients in two groups.

Indexes	CHD group (n = 157)	Non-CHD group (n = 185)	P value
Gender (male/female)	120/37*	104/81	P < 0.001
Type of chest pain (typical/atypical)	138/19*	123/62	P < 0.001
History of myocardial infarction (n/%)	48/30.6*	10/5.4	P < 0.001
History of hypertension (n/%)	99/63.1*	75/40.5	P < 0.001
History of hyperlipidemia (n/%)	28/17.8	36/19.5	0.701
History of diabetes (n/%)	42/26.8	35/18.9	0.084
History of smoking (n/%)	65/41.4*	51/27.6	0.007
History of alcoholism (n/%)	1/0.6	2/1.1	0.999
Family history of CHD (n /%)	3/1.9	5/2.7	0.629
Pathological Q wave (n/%)	35/22.3*	5/2.7	P < 0.001
ST-T changes (n/%)	35/22.3*	13/7.0	P < 0.001

### Comparison of quantitative data between CHD group and non-CHD group

The left ventricular ejection fraction (LVEF), anaerobic threshold heart rate, peak VO_2_/kg, peak VO_2_%pred, and OUES in the CHD group were statistically lower then those in the non-CHD group (P < 0.05). The age and VE/VO_2_ slope in the CHD group were higher than those in the non-CHD group, and the differences were statistically significant (P < 0.05). There were no significant differences in other indexes (Table 4).

**Table 4:** Comparison of qualitative data between two groups.

Indexes	CHD group (n = 157)	non-CHD group (n = 185)	P value
Age (year)	60.64 ± 7.77*	58.01 ± 9.48	0.005
Exercise time (min)	5:56 ± 1:44	5:41 ± 1:32	0.173
Indicators at rest			
Respiratory exchange rate	0.82 ± 0.09	0.81 ± 0.09	0.307
Heart rate (bpm)	72.73 ± 12.79	74.26 ± 11.18	0.238
Systolic pressure (mmHg)	137.68 ± 19.31	135.17 ± 18.45	0.222
Diastolic blood pressure (mmHg)	88.23 ± 12.43	88.97 ± 12.28	0.582
Anaerobic threshold indicators			
Respiratory exchange rate	0.84 ± 0.09	0.84 ± 0.10	0.673
Oxygen uptake (L/min)	0.85 ± 0.23	0.89 ± 0.28	0.155
Minute ventilation (L/min)	23.35 ± 6.38	22.14 ± 5.79	0.066
Power (W)	62.84 ± 20.15	61.18 ± 19.52	0.441
Heart rate (bpm)	101.09 ± 13.23*	104.28 ± 14.39	0.035
Metabolic equivalents	3.32 ± 0.72	3.33 ± 0.70	0.857
Oxygen pulse (ml/beat)	8.43 ± 2.09	8.51 ± 2.44	0.725
Indexes at peak			
Oxygen uptake (L/min)	1.24 ± 0.35	1.28 ± 0.35	0.272
Oxygen uptake in kilograms of body weight (ml/kg/min)	16.50 ± 4.08*	17.66 ± 4.15	0.010
Percentage of oxygen uptake to the predicted value (%)	64.31 ± 14.24*	69.95 ± 15.42	0.001
Minute ventilation (L/min)	38.95 ± 11.87	36.99 ± 11.75	0.127
Power (W)	99.46 ± 30.10	95.17 ± 31.09	0.197
Heart rate (bpm)	123.50 ± 17.09	125.68 ± 17.96	0.253
Metabolic equivalents	4.86 ± 1.24	4.79 ± 1.09	0.595
Oxygen pulse (ml/beat)	10.10 ± 2.86	10.28 ± 2.83	0.579
Systolic pressure (mmHg)	198.64 ± 24.17	194.75 ± 25.82	0.154
Heart rate reserve (bpm)	35.60 ± 17.16	35.26 ± 17.82	0.861
Physiologic dead space/tidal volume	0.24 ± 0.05	0.24 ± 0.05	0.413
Oxygen uptake efficacy slope (ml/min/W)	9.22 ± 2.54*	9.83 ± 2.16	0.017

### The diagnostic value of single-factor index in CHD

**Figure 5:**
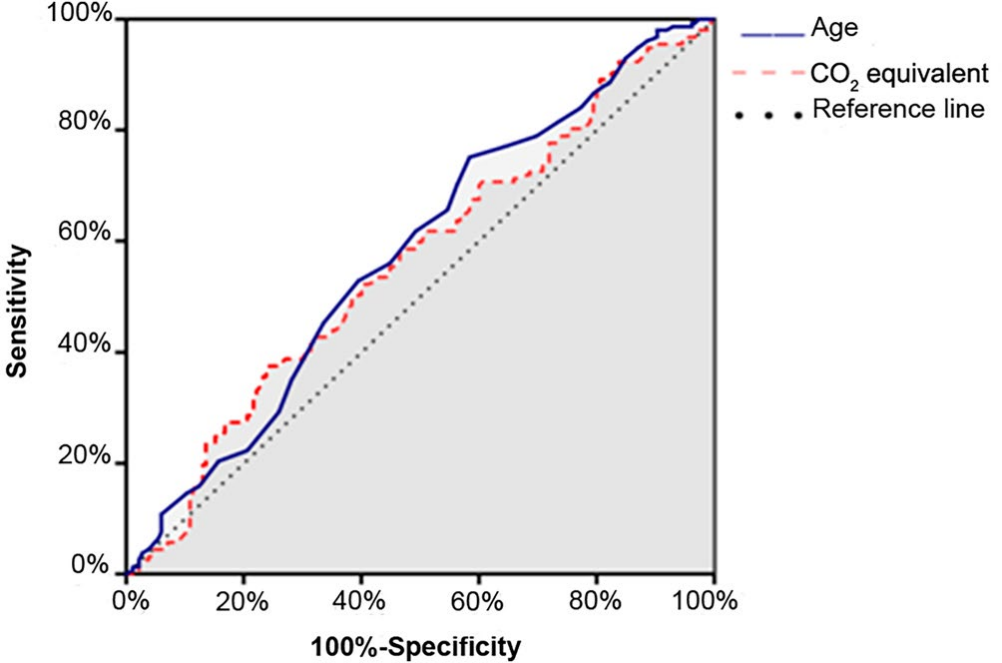
ROC curve of carbon dioxide ventilation equivalent (VE/VCO_2_) slope and age.

ROC curves were plotted (Fig. 5), and AUC_age_ = 0.579 (95% CI 0.518–0.639, P = 0.012) and AUC_CO2_ equivalent = 0.563 (95% CI 0.502–0.624, P = 0.046) were measured. The diagnostic sensitivity and specificity of VE/VCO_2_ slope were 37.6% and 75.7%, the positive likelihood ratio (+ LR) = 1.547, negative likelihood ratio (-LR) = 0.824. Age diagnostic sensitivity was 75.2%, specificity was 41.6%, + LR = 1.288, -LR = 0.596. The Youden index was 0.133 and 0.168, and the corresponding to cut-off values of 29.385 and 56.5, respectively.

### Results of multivariate analysis

#### Establishment of predictive models

The prediction equation was established as PTP_cardiopulmonary_ = 1/(1 + exp[−(−2.337 + 0.029 × age-0.013 × age × gender + 0.010 × age × smoking + 0.920 × typical angina pectoris + 1.257 × history of myocardial infarction + 0.793 × history of hypertension + 1.283 × pathological Q waves + 0.755 × ST-T changes-0.097 × peak oxygen pulse)]. PTP_cardiopulmonary_ was the pretest predicted probability of developing CHD, and the risk factor variables in the equation were assigned values (0 for none, 1 for yes, 0 for male, 1 for female). The variance expansion coefficients for age, age × gender, age × smoking, typical angina pectoris, history of myocardial infarction, history of hypertension, pathological Q waves, ST-T changes, and peak oxygen pulse were: 1.127, 1.901, 1.338, 1.077, 1.490, 1.082, 1.447, 1.173, 1.514, all less than 2. The tolerances were 0.888, 0.526, 0.748, 0.928, 0.671, 0.924, 0.691, 0.853, and 0.660, which were all greater than 0.1, respectively. Suggesting that there was no serious multicollinearity in the equation factors.

#### Distinctness and calibration of predictive models

A total of 102 patients were correctly classified in the CHD group, and 148 patients in the non-CHD group were correctly classified, with a sensitivity and specificity of 65% and 80%, respectively. The consistency parameter (C-statistic) was 0.731. Hosmer–Lemeshow goodness-of-fit test χ^2^ = 8.622, p = 0.375, suggesting that the constructed model has good discrimination and calibration for CHD. The Youden index of the model was 0.464, corresponding truncation value was 55.4%, at which time the sensitivity was 60% and the specificity was 87%. A line plot of the actual observed values versus the model predicted values is plotted (Fig. 2).

### Validation of model effectiveness

The ROC curves of the three models for predicting CHD were plotted with PTP as the test variable and ICA outcome as the status variable (Fig. 4), and the measured AUC_cardiopulmonary_, AUC_DCS_, and AUC_UDFM_ were 0.797 (95% CI 0.696–0.897), 0.774 (95% CI 0.669–0.880), and 0.716 (95% CI 0.603–0.829).

## Discussion

Early diagnosis and treatment of CHD are of great significance in improving patients' quality of life and long-term survival rate^1^. The current clinical tests for CHD have their limitations. Although ICA is used the "gold standard" for diagnosis, it is also subjective to visual inspection, invasive operation, and risk of complications. Foreign guidelines^3–6^ have implemented DCS and UDFM models based on European and American populations, which focus on symptoms, medical history, and ECG, and there is evidence-based evidence^7,13,14^ that the accuracy of these two models has decreased to varying degrees in the Asia–Pacific population. Compared to invasive tests, the non-invasive objectivity of CPET has obvious advantages, as it can combine the circulatory and respiratory systems to assess the overall cardiopulmonary function during exercise, perform repeatable quantification of cardiopulmonary function and provide prognostic stratification. Previous studies have demonstrated that peak oxygen uptake, peak oxygen pulse, anaerobic threshold and VE/VCO_2_ slope in CPET indicators can be used as risk predictors of CHD^10,12,15^. Pryor et al. concludedthat the overall risk of multiple risk factor interactions is stronger than the sum of single factor effects^16,17^. For example, there is an interaction between age and gender. Estrogen can improve vascular endothelial function. The premenopausal coronary arteries in female are protected by estrogen regulation, and the incidence of CHD is lower than that of male, and the significant decrease in estrogen after menopause leads to the same incidence rate as male. The differences in age, gender, and smoking history between the CHD and non-CHD groups in this study were statistically significant (P < 0.05), but the significance was weaker in the multifactorial analysis than the two composite indicators of age × gender and age × smoking, which was also indirectly evidenced. Progression of coronary calcification also increases atherosclerotic cardiovascular disease (ASCVD) events by 4- to sevenfold with age^18,19^.

Various PTP models include different indicators, and their clinical efficacy is also different. The previous Framingham risk score (FRS) model included seven CHD risk factors: age, blood pressure, TC, HDL-C, LDL-C, history of diabetes, and smoking history, and the overall predictive accuracy of the FRS for male and female was obtained after 10 years of follow-up, with a C-statistic of 0.69 and 0.72, respectively^20^. In this study, CPET indicators were included in the model multivariate analysis, and multiple logistic regression was used to establish the model equation as PTP_cardiopulmonary_ = 1/(1 + exp[−(−2.337 + 0.029 × age-0.013 × age × gender + 0.010 × age × smoking + 0.920 × typical angina pectoris + 1.257 × history of myocardial infarction + 0.793 × history of hypertension + 1.283 × pathological Q waves + 0.755 × ST-T changes—0.097 × peak oxygen pulse)], of which, PTP_cardiopulmonary_ is the probability of developing CHD predicted by the constructed model. The constructed model adds consideration of composite indicators, ECG, and medical history compared to the UDFM model, and two additional markers, peak oxygen pulse and history of hypertension was added in the the constructed model compared to the DCS model. The model AUC was slightly better than the DCS and UDFM models (0.780 vs 0.757 vs 0.699), and the C-statistic reached 0.731, indicating that the PTP model combined with the CPET index had better clinical differentiation ability for CHD.

The main reason for the improved accuracy of the constructed model is the inclusion of peak oxygen pulse as a characteristic index. The oxygen pulse is obtained by dividing VO_2_ by the synchronized heart rate, that is, VO_2_/HR. Reflects the heart's ability to deliver oxygen per beat. Ganesananthan et al.^12^ found that oxygen pulse curves can detect the severity of myocardial ischemia and predict the efficacy of interventional therapy in patients with monovascular lesions. As the exercise power increases during cardiopulmonary exercise, the oxygen pulse increases. When the oxygen pulse curve shows a low flat line phenomenon, that means the existence of oxygen pulse curve plateau, which is common in patients with ischemic heart disease. In this study, the stepwise regression analysis method was used to conduct multiple modeling comparisons for CHD-related factors. and the model with the best effectiveness only retained the peak oxygen pulse in the CPET index. The decrease in the value indicates that the extreme exercise leads to myocardial ischemia-induced cardiac output obstruction and an imbalance in oxygen supply induced by incremental body load, suggesting that the subject may have cardiac insufficiency. The peak oxygen pulse index is also significantly correlated with the oxygen pulse curve platform, and both are oxygen pulse curve-related description indicators. Munhoz et al. studied 87 patients who underwent both stress myocardial perfusion imaging and CPET and found that patients with a transient coronary perfusion deficit during extreme exercise had a lower peak oxygen pulse than patients in the normal group (12.8 ± 3.8 ml/beat vs. 16.4 ± 4.6 ml/beat)^20^. In this study, the differences in the peak oxygen pulse index between groups was not statistically significant in the univariate analysis (P = 0.579), which is consistent with previous studies, this may be limited by the small sample size or due to the confounding factors that weaken or even mask the association between peak oxygen pulse and CHD. After excluding the masking effect of related factors in multivariate analysis, it showed a strong correlation with CHD (P = 0.079). Peak oxygen pulse, as a protective factor for CHD, has some predictive diagnostic value for CHD. Combining it with the PTP model can increase the overall assessment of the model on cardiopulmonary blood supply loading capacity, thereby improving the clinical effectiveness of the model.

In order to better test the clinical effectiveness of the constructed model, 80 patients with suspected chest pain other than the constructed sample were randomly selected for effectiveness validation in this study, and found that the clinical effectiveness of the constructed model was also slightly better than that of the DCS and UDFM models (0.797 vs. 0.774 vs. 0.716), which demonstrates to some extent the better generalizability of the constructed model. However, due to the small sample size, all of them could not be included in the final model equation. As the database continues to be expanded and updated, there will be stronger evidence to use it as a risk marker, and the accuracy of the updated model may be further improved, and with the gradual increase of the subsequent validation sample, there will be more evidence-based basis to support the CHD pre-test probability model established combined with CPET indicators.

## Conclusions

The pre-test probability model of CHD combined with CPET indexes is good for the differentiation and calibration of CHD.

## Data Availability

All data generated or analyzed during this study are included in this published article.
