# Author Correction: Establishment and effectiveness evaluation of pre-test probability model of coronary heart disease combined with cardiopulmonary exercise test indexes

**DOI:** 10.1038/s41598-023-45892-9

**Published:** 2023-10-30

**Authors:** Si Xu Liu, Sheng Qin Yu, Kai Jing Yang, Ji Yi Liu, Fan Yang, Ye Li, Chang Li Yao, Guang Sheng Zhao, Feng Zhi Sun

**Affiliations:** 1 Heart Center, Affiliated Zhongshan Hospital of Dalian University, No. 6 Jie Fang Street, Zhongshan District, Dalian, 116001 Liaoning Province China; 2 Minimally Invasive Interventional Diagnosis and Treatment Center, Affiliated Zhongshan Hospital of Dalian University, No. 6 Jie Fang Street, Zhongshan District, Dalian, 116001 Liaoning Province China

Correction to: *Scientific Reports* 10.1038/s41598-023-41884-x, published online 29 September 2023

In the original version of this Article Guang Sheng Zhao was incorrectly affiliated with ‘Heart Center, Affiliated Zhongshan Hospital of Dalian University, No.6 Jie Fang Street, Zhongshan District, Dalian 116001, Liaoning Province, China’. The correct affiliation is listed below.

Minimally invasive interventional diagnosis and treatment center, Affiliated Zhongshan Hospital of Dalian University, No.6 Jie Fang Street, Zhongshan District, Dalian 116001, Liaoning Province, China.

The original Article has been corrected.

